# Advances in the Elimination of Viral Hepatitis in Mexico: A Local Perspective on the Global Initiative

**DOI:** 10.3390/pathogens13100859

**Published:** 2024-10-01

**Authors:** Gerardo Santos-López, Arturo Panduro, Francisca Sosa-Jurado, Nora A. Fierro, Rosalía Lira, Luis Márquez-Domínguez, Marco Cerbón, Nahum Méndez-Sánchez, Sonia Roman

**Affiliations:** 1 Laboratorio de Virología, Centro de Investigación Biomédica de Oriente, Instituto Mexicano del Seguro Social, Metepec 74360, Mexico; sosajurado@hotmail.com (F.S.-J.); lumardo80@gmail.com (L.M.-D.); 2 National Network of Viral Hepatitis Researchers, Mexico City, Mexico; apanduro53@gmail.com (A.P.); noraalma@iibiomedicas.unam.mx (N.A.F.); drarosalialira@gmail.com (R.L.); mcerbon85@yahoo.com.mx (M.C.); nmendez@medicasur.org.mx (N.M.-S.); 3 Department of Genomic Medicine in Hepatology, Civil Hospital of Guadalajara, Fray Antonio Alcalde, Health Sciences Center, University of Guadalajara, Guadalajara 44280, Mexico; 4 Department of Immunology, Instituto de Investigaciones Biomédicas, Universidad Nacional Autónoma de México, Mexico City 04510, Mexico; 5 Unidad de Investigación Biomédica Oncológica Genómica, Hospital Gineco Pediatría 3A, OOAD Cd Mx Norte, Instituto Mexicano del Seguro Social, Mexico City 07760, Mexico; 6 Facultad de Química, Universidad Nacional Autónoma de México, Mexico City 04510, Mexico; 7 Liver Research Unit, Medica Sur Clinic & Foundation, Mexico City 14050, Mexico

**Keywords:** viral hepatitis, HBV, HCV, HEV, viral hepatitis elimination

## Abstract

Viral hepatitis (A–E) presents a major global health challenge. In 2015, the World Health Organization (WHO) launched an initiative to eliminate viral hepatitis, with the aim of reducing new infections by 90% and deaths by 65% by 2030. Mexico is one of 38 focus countries identified by the WHO, collectively accounting for 80% of global infections and deaths. While hepatitis B and C are commonly diagnosed in Mexico, routine diagnosis for hepatitis D and E is lacking, with no specific epidemiological data available. In 2020, Mexico implemented the National Hepatitis C Elimination Program, focusing on preventing new infections, reducing complications like cirrhosis and hepatocellular carcinoma, ensuring access to treatment, and improving patient care. However, this program has not been extended to hepatitis B and E. Addressing the challenges of viral hepatitis control in Mexico requires increased resource allocation, expanded diagnosis, vaccination for hepatitis A and B, and treatment coverage for hepatitis B and C, along with multisectoral engagement. This work provides an overview of Mexico’s response to the global initiative, highlighting its progress, challenges, and areas of opportunity.

## 1. Introduction

Viral hepatitis (A–E) represents a significant global public health challenge due to its morbidity, mortality, and socioeconomic consequences. Based on 2015 statistics, the WHO member states approved the Global Health Sector Strategy (GHSS) for viral hepatitis in May 2016, with the intention of reducing new cases by 90% and deaths by 65% by 2030 [1]. The groundwork for WHO actions was laid during the 2010 63rd World Health Assembly, which declared July 28 World Hepatitis Day [2]. By adopting this global strategy, participating nations must strengthen public health efforts at all levels, including case detection, treatment, education, vaccination, and blood reserve surveillance.

Five distinct viral species are the primary causes of acute or chronic hepatitis. They are called hepatitis viruses (HV) and are classified as A, B, C, D, and E. Each virus follows specific patterns regarding their transmission route, establishment in the host, and acute or chronic manifestation. HAV (family *Picornaviridae*) and HEV (family *Hepeviridae*), both RNA viruses, are transmitted via the fecal–oral route, which includes the consumption of contaminated food and water, as well as by person-to-person contact. In contrast, HBV (family *Hepadnaviridae*), a DNA virus; HCV (family *Flaviviridae*), an RNA virus; and HDV (family *Kolmioviridae*), an RNA virus, are transmitted parenterally, primarily through blood transfusions or through the sharing of intravenous drug use equipment. Perinatal transmission can also occur, and sexual transmission is well documented in the case of HBV [3,4].

Due to their high impact, HBV and HCV have been identified as crucial targets for reducing the incidence of liver diseases, particularly chronic liver disease [5]. Nearly 90% of neonates and up to 5% of adults infected with HBV develop chronic infection, whereas 75–85% of HCV patients progress to chronic disease, increasing the risk of severe complications like liver cirrhosis and hepatocellular carcinoma [6,7].

However, other equally significant viral types have been eclipsed by the interest in HBV/HCV. Among these, the emerging hepatitis E virus (HEV), initially considered a poverty predictor, is a major problem in high-income regions. First thought to be self-limiting, HEV may cause persistent infections worldwide due to its diverse propagation (enterically by feces from mother to fetus, via blood derivatives, and between people and zoonotically), particularly in patients with compromised immune systems [8,9]. Globally, populations at risk of severe forms have been characterized: chronic liver disease patients are at risk of liver failure; pregnant women can present fulminant hepatitis; and immunosuppressed patients, especially those with solid organ transplants (SOT), are at risk for chronic hepatitis and rapid progression to cirrhosis [10]. Numerous extrahepatic symptoms of HEV reinforce the idea that infection is a systemic illness, not just a liver disease [9,10,11]. Likewise, hepatitis A virus (HAV) and hepatitis delta virus (HDV) infections, which are directly and indirectly vaccine-preventable, respectively, are often underestimated [12,13]. Elimination of HAV faces challenges due to the immunity gaps that exist in high-HAV-endemicity, low-resource areas, where natural immunity from prior infection during childhood has been achieved, or in countries where universal mass vaccination has been installed, causing higher susceptibility to hospitalizations or deaths in adolescents/adults who present with symptomatic, severe disease [14,15]. At-risk adults, including men who have sex with men (MSM), those living with human immunodeficiency virus (HIV), people facing homelessness, and injection and non-injection drug users need access to HAV vaccination coverage [15]. Likewise, eliminating HDV is challenging, both in endemic and non-endemic regions, due to subdiagnosis, which is attributable to a lack of robust epidemiological estimations, low awareness and public health resources, methodological issues, and ineffective treatments [16,17,18].

The global burden of disease from viral hepatitis B and C is high. About 300 million individuals are infected with HBV or HCV, with 6000 new infections daily. Also, the WHO reported a 20% increment from 1.1 million in 2019 to 1.3 million viral hepatitis deaths in 2022, of which 87% of fatalities were due to B and 13% to C [19]. According to WHO estimates, in 2019, over 150 million individuals were infected with HAV [20]. In contrast, the global burden of HDV and HEV is unknown. In the former, there is significant controversy surrounding the frequency of HBV-HDV superinfection [13]. Estimates are that it affects from 12 million to 72 million people who test positive for HBsAg globally [21]. A recent modeling study from the Polaris Observatory for HDV has underscored several factors that hinder the determination of the global burden of HDV [18]. As for HEV, mathematical modeling has predicted that HEV-gt1 and -gt2 produce 20.1 million new infections in Asia and Africa, with 3.4 million acute hepatitis E cases and 70,000 deaths in some areas of these continents. Unfortunately, these estimates exclude extrahepatic cases and the impact of HEV-gt3- and -gt4-related chronic disease. Therefore, the accurate picture of HDV and HVE infections is still incomplete [8,18].

The scarcity of economic resources, diagnostic technologies, medications, and policies of varying effectiveness are likely to lead to inconsistent outcomes in the viral hepatitis elimination efforts of many nations. The COVID-19 pandemic has exacerbated these disparities, particularly in low- and middle-income countries. The delay in economic, social, and health recovery has impacted viral hepatitis programs and other public health objectives in the short and medium term. Consequently, the transmission and mortality rates pose significant challenges to the global eradication of viral hepatitis by 2030.

In the case of Mexico, the initial commitment to the GHSS plan occurred in 2016 with the publication of its first document aligning with this strategy outlining the aims to curb the morbidity and mortality due to HCV through health promotion, prevention, diagnosis, and treatment initiatives targeting high-risk populations [22]. However, it was not until July 2020 that Mexico officially announced its inaugural National Hepatitis C Elimination Program [23]. As part of the National Network of Viral Hepatitis Researchers, we have followed with great interest the unfolding of the strategic actions, acknowledging the advances and evaluating the limitations that could jeopardize the success of reaching the 2030 goal. Therefore, this work provides a basic overview of viral hepatitis and Mexico’s local response to the global initiative, aiming to identify the progress, challenges, and potential opportunities contributing to the global effort of viral hepatitis elimination.

## 2. Materials and Methods

This article was constructed as a narrative review, incorporating relevant scientific literature obtained from PubMed (https://pubmed.ncbi.nlm.nih.gov/, accessed on 1 July 2024) and Scielo (https://www.scielo.org/, accessed on 1 July 2024). The review was based on the following criteria: updated studies on hepatitis A–E in Mexico covering epidemiological aspects, diagnosis, treatment, vaccination, and other related topics. Official documents from the Mexican government that establish guidelines for the national viral hepatitis elimination program were also included. Considering the dynamic nature of viral hepatitis elimination efforts both globally and in Mexico, we prioritized finding updated information and official reports from the WHO (https://www.who.int/, accessed on 1 July 2024) and the Mexican Ministry of Health (https://www.gob.mx/salud, accessed on 1 July 2024). These sources provided access to periodic publications such as the WHO’s *Global Hepatitis Reports* and the *Bulletin of the National Program for the Elimination of Hepatitis C* in Mexico.

The research group identified key aspects to highlight, leading to the development of sections that outline the current epidemiological situation of viral hepatitis in Mexico. In addition to addressing the general population, we focused on specific at-risk groups, including drug users, sex workers, and pregnant women, given their heightened risk of transmission or health complications. We paid particular attention to the availability of diagnostic tools and treatments within the framework of the hepatitis elimination program. Furthermore, we proposed recommendations and areas for improvement to enhance the effectiveness of the program. The authors, all members of the National Research Network on Viral Hepatitis, contributed to writing and discussing specific sections based on their diverse research backgrounds and expertise in different types of viral hepatitis. Due to the collaborative nature of this group, the final version of the article was thoroughly reviewed and discussed by all the authors.

## 3. The Seroepidemiological Landscape of Viral Hepatitis in Mexico

**Figure 1:**
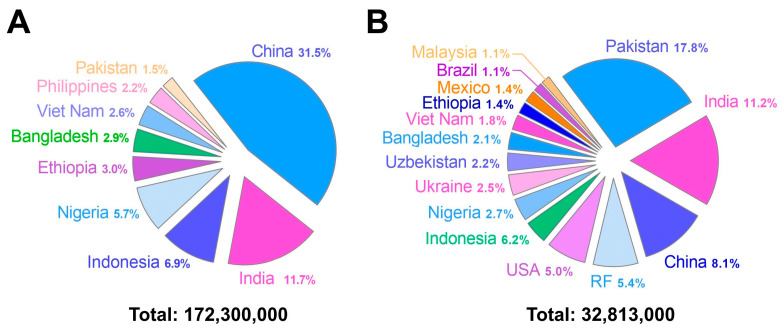
Countries contributing to two-thirds of the global cases of hepatitis B (panel (**A**)) and hepatitis C (panel (**B**)). Each panel shows the proportional distribution of cases by country. The total number of prevalent cases represents 67% (172.3 million out of 254 million) of the total for hepatitis B and 65% (32.81 million out of 50 million) for hepatitis C. Source: OMS [19].

Mexico is one of the 38 countries in the WHO that are focusing on the viral hepatitis response. These countries collectively account for 80% of viral hepatitis infections and deaths. According to the 2022 update, Mexico ranks 35th on the list of these 38 countries, with 0.04% of the total hepatitis B burden, a relatively low figure compared with countries at the top of the list, such as Ethiopia (3%), Nigeria (5.7%), Indonesia (6.9%), India (11.7%), and China (31.5%). Mexico is also among the 15 countries covering two-thirds of the global burden of hepatitis C, accounting for 1.4% of the total burden. Although this figure is relatively low, Mexico ranks 13th on the list of these 15 countries, with the top 5 countries having a significantly higher proportion of cases: the United States (5%), the Russian Federation (5.4%), China (8.1%), India (11.2%), and Pakistan (17.8%) [19]. Based on these data, Figure 1 separately illustrates the countries that account for two-thirds of all hepatitis B (panel A) and hepatitis C (panel B) cases worldwide.

Several national agencies report on the prevalence of viral hepatitis in Mexico, each with their own statistical methodology; these include the General Office of Epidemiology (Direccion General de Epidemiología; DGE in Spanish), the National Institute of Statistics and Geography (Instituto Nacional de Estadísticas y Geography; INEGI in Spanish), and the National Institute of Public Health (Instituto Nacional de Salud Pública; INSP in Spanish), as well as national surveys such as the National Health and Nutrition Survey (Encuesta Nacional de Salud y Nutricion; ENSANUT in Spanish).

Mexico, traditionally classified as a region with a high prevalence of HAV infection, is currently experiencing an epidemiological change. Nevertheless, scientific publications on the burden associated with HAV are scarce. In a study analyzing the General Office of Epidemiology data, the authors revealed a decrease to 6500 annual cases in 2017 from over 12,000 cases in 1994 [24]. However, during this study period, children between the ages of 1–14 comprised the most cases. In another observational, retrospective database analysis from the National Epidemiological Surveillance System (January 2000 to December 2019), the average annual incidence rate of HAV cases was 14.7 per 100,000 persons [25]. The age group of children between 1 and 9 years old had the highest average incidence rate each year, which was 47.8 per 100,000 persons. Thus, both studies underscore the high incidence of HAV during childhood in Mexico.

The 2018 ENSANUT, which employed probabilistic sampling across the country’s adult population, revealed a prevalence of HBV surface antigen (HBsAg) of 0.51% (95% CI 0.19–2.33%) [26]. Similarly, in this study series, the prevalence of anti-HCV antibodies in Mexico was 0.38% (95% CI 0.24–0.59%) [27]. Furthermore, the Polaris Observatory published a global study on the impact of hepatitis C, modeling available data to propose possible scenarios for eliminating viral hepatitis worldwide. Their research calculated a prevalence of 0.6% in the Mexican population for 2020 [28].

However, most of these agencies do not perform a breakdown analysis by subpopulations nor do they consider several factors that influence the prevalence rate, giving the false impression of low HBV and HCV infection rates in Mexico. However, using meta-analysis, it has been reported that HBV infection ranges from 2% to 12%, depending on the risk group, which represents at least 15 million people infected. In contrast, a systematic review spanning 2008 to 2019 reported a weighted seroprevalence of HCV that ranges from 1.35% (95% CI 1.21–1.48%) in the general population to up to 36.79% (95% CI 36.76–36.81%) in high-risk groups [29]. Furthermore, local studies in low-income populations have reported a higher seroprevalence of 1.0% and 3.9% for HBV and HCV, respectively [30].

On the other hand, it is important to emphasize that Mexico’s population comprises native and admixed individuals, and a high proportion of the former group shows evidence of occult hepatitis B (OBI), which is characterized by viral DNA in the liver of HBsAg-negative individuals, with or without detectable viral DNA in the blood. A critical limitation in this context is the reliance solely on HBsAg as the serological marker for HBV infection, without testing for anti-HBc or nucleic acids. As a result, OBI may go undetected in many individuals, leading to the false perception that HBV infection in Mexico is relatively low [31].

Another concerning situation is that incidents mandatorily notified by the national Ministry of Health comprise only patients with hepatitis A, B, and C. In contrast, patients with suspected hepatitis D or E may fall in the category of “other hepatitis”; hence, lacking a precise diagnosis. Therefore, there is no explicit epidemiological data for these two viruses in Mexico, and information comes from studies by independent research groups. In this sense, seroepidemiological data related to specific geographic regions have been reported. Two earlier HDV research studies, one conducted in a southeastern rural community revealed 42% (50/119) of anti-HDV antibodies among HBsAg-positive individuals [32] while another in northwest Mexico reported 4% [33]; however, there has not been further follow-up in nearly 50 years. On the other hand, variable frequencies between 0% and 57.5% have been reported for HEV, underscoring a differential distribution of the virus that may be related to distinct transmission sources [34].

In a 2005 epidemiological prediction study, it was estimated that by 2050, two million chronic cases would be present in Mexico, and hepatocellular carcinoma would become the third leading cause of liver-related mortality [35]. According to data from 2022, liver diseases rank as the fourth leading cause of death in Mexico [36]. It has been foreseen that the etiology profile related to liver damage is shifting from viral hepatitis toward steatohepatitis due to better treatments and vaccination coverage. However, the lack of systematic diagnosis of viral hepatitis in Mexico undermines the possibility of determining the attributable fraction of these etiological agents to the burden of end-stage cirrhosis and hepatocellular carcinoma (HCC). For example, one study estimated that 50% of cirrhosis cases are caused by hepatitis B and C viruses [37], whereas in another, viral hepatitis was reported in third place [38].

Nonetheless, underestimating HBV will hinder the likelihood of its future elimination. Therefore, further prospective and retrospective studies are required to establish the main causes of liver damage in patients with clinical hepatitis, as well as the implementation of a robust public health registry of these cases. On the other hand, there are few studies on pregnant women, a significant public health concern due to the impact on mother–child binomial health.

## 4. Viral Hepatitis in Pregnant Mexican Women: Clinical Aspects and Difficulties in Its Treatment and Eradication

A recent study performed at the National Institute of Perinatology (INPer) reported a prevalence of 0.22% for viral hepatitis in pregnant Mexican women, with a non-significant predominance of HCV over HAV and HBV [39]. This study reported that HBV and HCV were screened at admission to the hospital and frequently at the first medical examination. In contrast, HAV was only tested in patients with hepatic symptomatology; however, screening for HAV and other viral hepatitis types, such as HEV and HDV, is currently not carried out at the INPer. It is believed that in Mexico, there is an underestimation of cases of viral hepatitis in pregnant Mexican women (0.22%), which could be related to the fact that, in this country, there is no regular screening for HEV and HDV infection. Regarding the clinical aspects of the disease, the same study reported that the clinical evolution of HAV, HBV, and HCV infection proceeded with similar symptoms as in non-pregnant patients [39]. Interestingly, in another study, acute HAV infection was more symptomatic and carried higher risks for mother and fetus than HEV infection [40]. Remarkably, histopathologic changes have been detected in all placentas, suggesting its participation as a vital tissue barrier against fetus infection. However, this topic remains to be explored more.

Concerning viral hepatitis eradication strategies in Mexico among pregnant women, success may be related to vaccination at birth, while the national HCV eradication program aims to treat the infection with direct-acting antivirals. The HBV strategy has had a positive impact on pregnant women and their children, reducing the risk of vertical transmission. The strategy against HCV is still at an early stage and is expected to be equally successful. For acute hepatitis, HAV, and HEV, programs promoting hand washing, clean food, and water supply are applicable as prevention strategies together with other programs such as vaccination. In conclusion, viral hepatitis during pregnancy requires the implementation of public health strategies for its elimination, the proper clinical management of the mother–child binomial, and the follow-up of them.

## 5. Molecular Epidemiology of Viral Hepatitis in Mexico

Among the ten genotypes (A–J) of the HBV, genotype H is the most predominant, accounting for nearly 70% of the total, followed by genotypes G, A, and D in another 25%; other genotypes have been reported in minimal proportions [41]. The presence of genotype H in Mexico is historical, with studies tracing the most common ancestor to Mesoamerican populations as far back as at least 4000 years [42]. This prolonged coexistence could explain the unique characteristics of the natural course and the epidemiological profile of HBV infection in the Mexican population. These characteristics include a high prevalence of anti-HBc antibodies, a low prevalence of HBsAg (likely due to a highly adaptive immune response), and a low viral load [43]. Overlapping with this profile is the presence of OBI, as mentioned before, among the general population, blood donors, native groups, and high-risk groups such as the HIV-positive population and MSM. However, due to the lack of comprehensive HBV marker testing, the fraction of overt or occult HBV attributable to the burden of HBV-related cirrhosis and HCC remains unclear. HCC in Mexico has not been fully documented, although estimations suggest a low prevalence due to distinct genetic and environmental factors, including the population’s adaptability to HBV genotype H [44]. Nonetheless, questions have been raised as to whether non-H genotypes such as F1b can result in liver carcinogenesis among native or admixed populations, as reported in other regions of the world, such as Alaska and Peru. These findings underscore the importance of molecular epidemiological studies to decipher the host–virus relationship and to comprehend the clinical intricacies of infection in Mexicans [43].

The molecular epidemiology of HCV is highly complex compared with HBV due to at least 70 subtypes existing within the seven genotypes identified to date. Molecular epidemiological studies of HCV sequences have detected the emergence of HCV subtype 3a in western and northeastern Mexico [29], which is primarily associated with parenteral transmission. Notably, the profile of HCV acquisition risk factors with the concomitant subtype has shifted over the years, as shown recently in a molecular evolutionary study [45]. This study revealed that HCV subtype 1b infection was common among women in their fifties and sixties with a history of gynecology- and obstetrics-related surgery or blood transfusion before 1994. However, as these women have either passed away or been cured, the frequency of this subtype has decreased. In contrast, the prevalence of HCV subtypes 1a and 3a has increased among men compared with women due to injection drug use. Furthermore, subtype 1a has shown clade diversification during its evolution, suggesting the dissemination of two distinct infection sources that may eventually lead to new subtypes or genotypes [45].

In the late 1980s, the first recognized virus outbreak in Latin America related to HEV was reported in Mexico, allowing HEV-gt2 to be described for the first time in the world; no molecular investigations on human HEV infections had been performed in Mexico until 2018, when the circulation of HEV-gt1 was confirmed in samples from pediatric patients with acute hepatitis [46,47]. Later, HEV-gt3 was reported from a retrospective analysis of samples from patients with chronic liver disease [48]. The reported viremia in blood donors emphasizes the need for HEV screening in Mexican blood banks to avoid the potential for virus transmission [49], since HEV screening via blood banks is not mandatory in the country. Moreover, chronic infection with HEV-gt3 has been recently identified among hemodialysis patients in Mexico, highlighting the importance of opportunely identifying the infection in this population [50]. Finally, HEV-gt3 has been identified in swine in the country, raising the alert for potential zoonotic infections [51].

Despite these initial efforts, further molecular studies are required for all hepatitis viruses, because they provide data on the geographic distribution and frequency of the specific genotypes, the tracking of the dissemination route, the antiviral efficiency, mutation emergence, and evolutionary analyses. Such data are relevant to link these features to the outcome of infection in patients with clinical hepatitis.

## 6. Viral Hepatitis Diagnosis in Mexico

A crucial aspect of Mexico’s current program is detecting infected individuals. Currently, this effort has been strengthened by the utilization of the CAPASITS system (Outpatient Center for the Prevention and Care of AIDS and Sexually Transmitted Infections), which are outpatient units established approximately 20 years ago for HIV prevention and care and which now offer free rapid tests for HIV, HCV, and syphilis to users. These centers, present in all states of the country, have expanded their coverage through mobile units, providing testing and training for healthcare personnel and the public [23].

According to the current guidelines, all healthcare facilities within their respective populations must integrate into the HCV screening system. Primarily, these screenings are predominantly conducted by facilities under the National Center for the Prevention and Control of HIV and AIDS (CENSIDA), the Mexican Institute of Social Security (IMSS), and the Institute of Security and Social Services for State Workers (ISSSTE). The general recommendation is that all individuals aged 18 and above undergo a rapid test to detect HCV. Any adult requesting the test should receive it, regardless of risk factors. Individuals who test positive are referred to public health services for confirmation and the determination of viral load through molecular tests. Treatment is then provided based on the patient’s virological and clinical status [52].

Although blood banks have historically been used to detect asymptomatic individuals, donor filters have led to fewer seropositive detections than in the general population [29,53]. Detecting infected blood donors has allowed many people to receive treatment in various Mexican health systems. However, while this increases the safety of blood reserves, rejecting donor candidates before conducting diagnostic tests reduces the opportunity to detect asymptomatic infected individuals. Establishing peripheral or mobile testing sites across the country increases the likelihood of detecting infected individuals and will consequently reduce infection transmission rates.

The diagnosis of hepatitis B is complex and involves the detection of viral nucleic acid and the use of various serological markers for viral antigens and antibodies. These complexities may hinder universal diagnosis, yet they are imperative as specific treatments, despite varying in efficacy, which depends partly on the detected markers [54]. It is, therefore, important to select tests according to the particular needs. The sensitivity of available tests has increased in recent years. For example, the detection limit of HBsAg in ELISA assays has improved from 0.05 IU/mL to 0.005 IU/mL. Nonetheless, the specificity of the commercial diagnostic kits regarding their reactivity in detecting the genotype H HBsAg requires further investigation [43]. The same may occur with anti-HB detection systems and the detection of antigenic variants of HBsAg. Nonetheless, nucleic acid detection systems have high sensitivity; some commercial systems can detect 100 copies/mL of HBV DNA, while viral RNA detection systems, currently only used in research, have detection limits of 10 copies/mL [55].

HBsAg in serum is crucial for defining infection; however, the complexity of HBsAg detection persists. An ongoing challenge, as mentioned before, is identifying OBI. Various approaches are being pursued to identify patients with OBI, but a universal solution still needs to be discovered, posing a risk of infection dissemination [56,57]. Serological diagnosis in blood banks in Mexico mandates safety studies through HBsAg detection; however, due to diagnostic complexities, it is advisable to incorporate the detection of at least one of the anti-HBc or anti-HB antibodies to broaden the coverage, especially for cases evading HBsAg detection, such as those with OBI, where it has proven to be a crucial marker in some instances.

Although Mexican health systems do not routinely detect HEV, scientific studies have underscored the presence of this pathogen in animal and human populations. A seroprevalence ranging between 0% and 57.5% in distinct human cohorts across the country has been reported, and more information is needed regarding the molecular detection of this virus in Mexico [34]. Moreover, since there is not yet a gold standard for detecting anti-HEV antibodies, distinct assays have been used in Mexico, all of which have differences in their detection limits, and the results are, therefore, not comparable. In that sense, even with very little molecular data reported, these findings confirmed the circulation of HEV in Mexico, supporting the fact that infection is still neglected in the country. Finally, HDV routine testing is part of a vicious cycle in which the lack of research in the field undermines the awareness that at-risk populations with chronic HBV infection could become co-infected with HDV.

## 7. Prevention of Viral Hepatitis in Mexico

Preventing and controlling viral hepatitis reduces public health concerns. Along with other sanitary measures, HAV, HBV, and, indirectly, HDV are controllable by vaccination, which has proven to be a cost-effective strategy for preventing these infections globally [58].

Improving hygiene, sanitary measures, access to clean water supply, and HAV vaccination prevents fecal–oral HAV transmission. HAV vaccines licensed since 1995 in the United States have been recommended by the WHO to be applied as part of universal mass vaccinations or catch-up schemes to avoid immunity gaps that lead to episodes or outbreaks that occur in regions transitioning from a high to an intermediate prevalence [58].

HBV vaccination, good healthcare, condom usage, and intravenous drug-user hypodermic needle exchange programs prevent HBV transmission. Minimizing the disease burden of these infections requires early detection and treatment, whereas effective medicines are needed to limit new case transmissions. The HBV vaccine was licensed in 1982 and is sold internationally. Infant HBV vaccinations provide 94–98% protection. The vaccine is based on the surface antigen of a genotype A2 virus, an adw serotype typical of genotypes A, B, F, G, H, and I, and it is widely distributed. Ayw, ayr, and adr, the other three major serotypes, may interact differentially with immune response components. Non-A genotypes may evade the vaccine-induced response, although they are associated with disorders without clinical significance [59]. The immune responses of vaccinated individuals to different virus genotypes and serotypes are influenced by antigenic variations, which can lead to failures in HBsAg detection. These variations involve a key epitope, known as the “a” determinant [59,60]. This data highlights the global antigenic diversity and underscores the need for studies on vaccine effectiveness.

In Mexico, HAV vaccination was introduced in 2008 to protect at-risk populations, and in 2013, a single dose was approved for children over one year of age. However, HAV vaccination is not mandatory, despite the number of HAV cases in children being nearly equal to the combined cases of HBV and HCV infections reported in adults (Table 1).

Given this data, the risk of severe disease in older individuals, and the high costs associated with hospitalizations due to complications, it has been recommended that universal mass vaccination programs be implemented [24,25]. Vaccination campaigns against hepatitis B were introduced in 1999 using the pentavalent whole-cell vaccine (diphtheria, pertussis, tetanus, hepatitis B, and *Haemophilus influenzae* type B). In 2007, the vaccination schedule was modified to include monovalent anti-hepatitis vaccines. The schedule for monovalent vaccines includes administration at birth, two months, and six months; for hexavalent vaccines, the schedule is at birth, 2 months, 4 months, 6 months, and 18 months. However, vaccination coverage is incomplete and discrepant. In 2023, a coverage of 83.3% was reported for the hexavalent scheme in children under one year of age, while others reported a coverage of 31.8% in adolescents [61]. However, it is essential to mention that HBV vaccines developed using international standards for genotypes A or D may not be equally effective in populations such as Mexico, which has the HBV genotype H epidemiological profile, as previously described [43]. Further studies are required to evaluate if anti-HB antibodies achieve protective levels.

**Table 1:** Relevant data on the status of viral hepatitis in Mexico and recommendations.

Type of Hepatitis	Annual Cases (2023)	Diagnostics	Vaccines/ Schedule	Treatment	Recommendations
A	4242 [62]	Yes	At 12 months [63] Not mandatory	Symptomatic [64]	Reduction of vaccination gap in adolescents and adults
B	618 [62]	Yes	Hexavalent/ Monovalent [63]	Entecavir/ Tenofovir [52]	Use of anti-HBc and NAT testing, use of DBS, expansion of vaccination coverage, access to treatment
C	3895 [62]	Yes	Null	Sofosbuvir/Velpatasvir [52]	Awareness campaigns, screening, and access to treatments
D	Not reported	No	Protected by vaccination against HBV	Pegylated interferon alpha [52]	Allocation of resources for research in high-risk groups
E	1892 * [62]	No	Null	Symptomatic [64]	Implementation of diagnostic testing

* Reported as “other viral hepatitis”; DBS—paper-based dried-blood sampling; [reference].

Regarding hepatitis C, no vaccine is currently available, although numerous research groups are actively working on its development [65]. Unlike hepatitis C patients, who have highly effective treatments available, there is no authorized treatment for hepatitis E; hence, global immunization is required [66]. An approved Chinese HEV vaccine has shown promise in recent African outbreaks, although it is not widely available [67]. A recombinant truncated capsid protein vaccination protects against acute hepatitis symptoms but not infection and is usually used to avoid outbreaks. Although it may prevent severe infection, the vaccine’s effectiveness and tolerability have not been studied in chronic liver disease and immunosuppressed individuals. This situation also occurs in gt4-dominant regions. Studying viral variants is crucial for developing protective immunization [68]. While progress has been made in vaccine development, significant gaps in coverage and effectiveness still need to be addressed, especially for vulnerable populations.

Mexico has a robust national vaccination program, which has generally resulted in high vaccination rates. However, regional variations and specific challenges may exist in certain areas. The country has a history of successful public health campaigns targeting various vaccine-preventable diseases, contributing to a generally positive perception of vaccines.

A recent study on COVID-19 vaccine acceptance found that 62.3% of respondents were willing to receive the vaccine, while 28.2% refused, and 9.5% expressed hesitation.

These data are significant due to the high levels of vaccine refusal and hesitancy, although the survey was conducted prior to the vaccine rollout [69]. The study does not explore the reasons behind these attitudes. It is important to note that these data may not reflect attitudes toward other vaccines, as the COVID-19 vaccines were impacted by an intense “infodemic”, which likely played a key role in shaping public opinion. In contrast, estimates from a year later showed the COVID-19 vaccination coverage to be at 85% among adults [70]. This suggests that public attitudes in Mexico are neither static nor uniform and are influenced by factors such as access to information and, most importantly, access to the healthcare system.

## 8. Management of Viral Hepatitis by Antiviral Treatment in Mexico: Strengths and Limitations

One of the most valuable aspects of the Mexican program is that diagnosis, medical care, and treatment are fully covered by the State and incur no cost for the user. However, according to data shared in the WHO report, coverage of these needs to be higher, but it remains low and requires greater attention for improvement. A limitation of Mexico’s national program is the inability to purchase or produce certain generic drugs, which are more economically accessible. For instance, in 2018, Gilead, the patent holder for hepatitis C drugs such as sofosbuvir, ledipasvir, and velpatasvir, granted voluntary licenses to certain companies for their distribution as generics in more than one hundred countries. However, some countries were excluded from this opportunity [71]. Similar circumstances have occurred with Abbvie, which signed a voluntary license with the Medicines Patent Pool (MPP) to manufacture and distribute the hepatitis C drugs glecaprevir/pibrentasvir in 96 low- and middle-income countries. The United Nations-supported MPP aims to promote access and facilitate medicine development [72]. Ideally, an agreement with the pharmaceutical companies to increase treatment coverage through reduced costs or expanded access to generics is necessary.

In Mexico, the State provides free hepatitis C treatment with direct-acting antivirals (DAAs), which target three viral proteins (NS3, NS5A, and NS5B) and are more than 95% effective at eliminating the virus. The efficacy of DAAs has been successfully tested in Mexican patients, confirming this high effectiveness rate [73]. Additionally, DAAs have been shown to eliminate the virus, improve liver health, and act as an adjuvant in treating extrahepatic manifestations. These include lymphoproliferative disorders such as low-grade marginal zone lymphoma, a type of non-Hodgkin’s lymphoma, mixed cryoglobulinemia, metabolic disorders, and cardiovascular risk [74]. In cases of treatment failure, clinicians can pursue alternative treatments based on individual circumstances [52].

Unlike HCV, complete elimination of or a cure for HBV cannot be generally achieved for patients. However, the current treatment aims to attain a functional cure, defined as the sustained loss of HBsAg and viral DNA in the blood with or without antibodies against HBsAg. There is no specific treatment for HEV, and no diagnosis of this virus prevails in the country. Therefore, the complexities involved in the diagnosis and treatment of HBV and HEV underscore the urgent need to formally establish national hepatitis B and E elimination programs in Mexico.

HAV infection, while often asymptomatic and self-limiting in children, can cause symptomatic and severe liver disease in older individuals, leading to hospitalizations. A recent study estimated an annual hospitalization rate of 5.8% and noted a rise in both the incidence of HAV infection and hospitalizations with complications in older age groups (10–64 years and above) [25]. Additionally, the annual mean mortality rate was calculated to be 0.44%, with 28.8% of deaths occurring in individuals aged 65 years and older. These cases can result in direct costs of up to MXN 382 million (approximately USD 21 billion). Therefore, preventive measures are necessary for these age groups.

Clinical studies indicate that chronic HDV infection represents the most severe and progressive form of viral hepatitis in humans. HBV co-infection with HDV is particularly concerning due to its rapid progression toward HCC and liver-related death [19]. HDV prevalence remains unknown in several world regions, even 40 years after its identification. Given the prevalence of HBsAg in Mexico, including HDV detection in prevalence studies is urgent, particularly in risk populations. As per established guidelines, suspected HBV-HDV co-infected patients who test positive for HBsAg should have their anti-HDV levels evaluated. Special consideration should be given to individuals with low or undetectable HBV-DNA levels but elevated ALT or AST levels [52]. Recently, the WHO highlighted HDV diagnostics as a priority innovation action in its 2024 Global Hepatitis Report, emphasizing the importance of HDV prevention programs [19].

## 9. Viral Hepatitis Elimination Program in Mexico

The National Hepatitis C Elimination Program, with guidelines published in 2020, was established as a priority program offering free medical care and medicines, targeting individuals without social security who are typically highly or very highly marginalized [23]. The central idea behind this initiative was to form a unified national response for preventing, treating, and eliminating hepatitis C. It operates under the auspices of CENSIDA, a decentralized body of the Mexican Ministry of Health responsible for implementing the national program for HIV/AIDS and the prevention and control of sexually transmitted infections [52].

According to the program’s central document, three priority objectives can be summarized as follows: (1) prevention of new infections and health promotion through mass media; (2) case detection and reduction of serious complications such as cirrhosis and hepatocellular carcinoma; and (3) ensuring access to optimal treatments and promoting treatment adherence, as well as enhancing the management and comprehensive care of patients [23]. The program specializes in marginalized populations with limited access to health services, providing free diagnosis, care, and treatment. A hepatitis B program with characteristics similar to hepatitis C has not yet been implemented in Mexico, although efforts are underway to establish one [41]. The latest update of the clinical guide on viral hepatitis within the Mexican health system emphasizes hepatitis B and hepatitis C [52]. Since there are no official epidemiologic data for hepatitis E, specific guidelines for its control are unavailable [34,75].

As of 2022, Mexico has reported a total of 117,412 HBV infections and 3927 deaths, while 678,258 HCV infections and 5736 deaths have been reported. The diagnostic coverage for HBV was 11.4%, which is comparable to that in Colombia (12.1%) but substantially lower than that in Peru (17%) and Brazil (34.2%). These countries are cited because they belong to the same region (Latin America) and are on the list of 38 focus countries. For HCV, the diagnostic coverage was 16%, which was greater than that in Colombia (12%) and Peru (5%) but significantly lower than that in Brazil (36%). Treatment coverage was barely 1% for HBV, similar to the countries mentioned above, with Brazil having the highest coverage at 3.6%. For HCV, treatment coverage was 4%, somewhat higher than for HBV but significantly lower than the 24% coverage achieved in Brazil [19].

In its biweekly report dated 20 May 2024, the Mexican Ministry of Health disclosed that between 1 January 2023 and 20 May 2024, a total of 674,554 screening tests for HCV detection were conducted, 15,077 of which yielded reactive results (2.2%) [76]. Additionally, the March 2024 bulletin revealed that up to that point, 36,619 individuals had tested positive for viral load. These individuals were subsequently referred for specialized medical care and tailored treatment. Sociodemographic data indicated that 42% of them had been or were currently incarcerated, 45% had used or were currently using intranasal substances, 47% had used injectable substances, and 4.6% were co-infected with HIV. This report showed that of the total rapid tests distributed, CENSIDA processed 83%, IMSS processed 15.5%, and ISSSTE processed 1.2% [77].

In a public report issued on the occasion of International Hepatitis Day in mid-2023, the Ministry of Health disclosed that since the inception of the program, over 2.3 million tests had been administered, resulting in treatment for 28,978 individuals infected with HCV, 22,748 of whom were successfully cured. This report also highlighted a significant expansion in diagnostic and care units from 42 to 629, along with training provided to 333,000 health professionals for the program implementation [78,79]. As of May 2024, screening tests that had been conducted on a total number of approximately 2.8 million individuals [76].

At this point, it is necessary to mention the discrepancy in the ENSANUT data, which calculates shallow prevalence values of hepatitis C in the Mexican adult population at 0.38% of anti-HCV [27], as already mentioned in the text. According to the most recent data from the program, in which rapid HCV tests were applied, 2.2% of the tests were positive [76]. This value may be overestimated, considering that rapid tests may have less specificity than conventional ELISA tests. Additionally, the ENSANUT sampling uses a randomized design throughout the country to minimize biases, whereas the current tests in the program, even if distributed nationally, may not achieve the same. However, other systematic reviews and meta-analysis studies provide data closer to the recent value of the elimination program, suggesting a prevalence higher in the general population, between 1% and 2%, and substantially higher in specific risk populations such as prison inmates, drug users, and dialysis patients [29,80,81]. Two other recent studies have also reported on the prevalence of HCV in Mexico, both using rapid tests. The first study, conducted between 2017 and 2019, included nearly 300,000 individuals from all states and reported a seroprevalence of 4.5% and an RT-PCR positivity rate of 3.2% [82]. The second study, spanning 2021 to 2022, involved over 75,000 people across 26 of Mexico’s 32 states, with a reported seroprevalence of 2.7% [83].

Resolving these discrepancies is crucial to better focus the sampling strategy, allocate funds, and improve the diagnostic infrastructure, health personnel, and the treatment and follow-up of patients. One proposal that can contribute to this process is the possibility of having open access to data, like the historical open data on influenza, COVID-19, and other respiratory or vector-borne diseases. The granularity of such data and parameters would greatly aid independent analysis and research, thereby enhancing the effectiveness of the hepatitis elimination program.

## 10. Challenges and Opportunities to Reduce the Impact of Viral Hepatitis in Mexico

An unavoidable challenge is the greater availability of public (and ideally private) resources to address pending aspects of the program. In low- and middle-income countries, competing emergencies across various national sectors often make allocating sufficient funds to each need difficult. Addressing this requires assessing the problem’s significance using data and appealing to the political will of decision makers.

In Mexico, the viral hepatitis elimination program remains incomplete. It primarily focuses on hepatitis C, which has a more significant impact than hepatitis B, E, and even D in the country. However, efforts are underway to establish a program that encompasses all types of hepatitis [41].

Increasing diagnostic coverage necessitates expanding the number of fixed and mobile units conducting tests and reaching deeper into the population. The most direct and efficient method for diagnosis, especially given its time advantage and the fact that highly specialized personnel are not required, is through the use of rapid tests. However, this alone is insufficient, as confirmation, along with genotype determination and viral load measurement, necessitates the use of molecular tests. Therefore, diagnosing diseases in marginalized and underserved rural populations poses a significant challenge due to their limited access to quality healthcare. One major obstacle to accurate diagnosis is the preservation and transportation of blood samples, which require a cold chain to maintain the integrity of target molecules such as nucleic acids and proteins until processed in reference laboratories. Emerging technologies such as paper-based dried-blood sampling (DBS) are promising for improving disease diagnosis in such populations. DBS, a simple, noninvasive blood collection method, can potentially be used in resource-poor healthcare settings, with samples processed later in appropriate laboratories for the delivery of results [84,85].

Additionally, samples collected via DBS can be transported and stored at room temperature, reducing cold-chain costs. A recent systematic review evaluated the utility of DBS tests in detecting HBV infections, demonstrating the simplicity and cost-effectiveness of using DBS samples with ELISA to detect HBsAg and its potential as an alternative sampling tool to improve HBV diagnosis in resource-limited settings [86]. Another recent study confirmed that dried samples are an alternative for HCV screening and antigen testing [87]. Implementing these DBS sampling techniques in Mexico presents an opportunity to strengthen the diagnostic capabilities for viral hepatitis.

A promising resource for increasing the diagnostic coverage is self-testing, particularly for high-risk populations, as recommended by the WHO. In 2021, the WHO published guidelines on self-testing [88]. By mid-2024, the WHO had prequalified a home-use test for the detection of anti-HCV antibodies, which uses a finger-prick sample and is specifically designed for non-specialist use [89]. In Mexico, self-testing remains underexplored, but given its advantages, it should be incorporated into hepatitis C elimination strategies. The documented experiences of other countries, with both saliva and blood samples, highlight its potential acceptance among users and the reliability of results [90,91].

This global initiative is inherently complex and requires engagement from multiple societal sectors. In Mexico, the initiative is centralized at the federal government level, necessitating active participation from other sectors, including health institutions in federal entities. Achieving these objectives demands concerted scientific, private, and governmental efforts. Scientists possess the knowledge and experience to develop new tests, treatments, and vaccines for hepatitis. At the same time, private companies have the financial resources and manufacturing capacity to bring these technologies to market. Collaboration between scientists, private companies, and the government can facilitate progress. Mexico’s experience during the COVID-19 emergency in developing mechanical ventilators and a vaccine through collaboration between the National Council of Humanities, Sciences, and Technologies (CONAHCYT) and the private sector demonstrates the potential for a similar collaboration in effectively achieving the global initiative’s objective of viral hepatitis elimination.

At this time, it is difficult to assess the level of public awareness regarding the availability of diagnosis and treatment options for hepatitis C. An effective way to increase awareness about the importance of hepatitis C testing is through mass media promotion, including radio, television, and social media. A notable example is the “Yo ya C” campaign, which roughly translates to “I know” or, in context, “I know about hepatitis C.” The campaign emphasizes that treatment is available to cure hepatitis C and ends with the call to action: “Inform yourself. Get tested” (https://www.gob.mx/censida/es/articulos/yo-ya-c-como-curar-la-hepatitis-c?idiom=es, accessed on 1 July 2024). While this is a valuable public education tool, its reach has been limited. Expanding or diversifying campaigns like this may be necessary to improve program coverage. To achieve this, it is likely necessary to forge alliances with the media to enhance the delivery of information to all strata and regions of the country.

## 11. Final Comments

The National Viral Hepatitis Elimination Program in Mexico marks a significant step in enhancing public health and mitigating the burden of viral diseases. Prioritizing the prevention, detection, and treatment of hepatitis B and C, especially among marginalized populations without social security, underscores the government’s commitment to elimination efforts. Currently, 2.8 million screening tests have been administered, and treatment has been provided to nearly 30,000 people infected with HCV. However, the process needs to be further expanded.

While the program focuses more on hepatitis C, the absence of formal hepatitis B and E programs highlights the need for expanded efforts to address these infections and to establish a national elimination plan. Improved case detection facilitated by the CAPASITS system and free rapid testing signifies progress, yet challenges persist, particularly in hepatitis B. While free hepatitis C treatments are available, coverage remains lower than in other Latin American countries.

Globally, Mexico is a priority country for the viral hepatitis response despite the relatively lower disease burden of hepatitis B and C. In the case of hepatitis E, the situation is even more complex. Its ability to persist infectiously under a variety of environmental conditions contributes to virus dissemination. Moreover, strategies to detect autochthonous HEV variants are needed to identify transmission sources and to prevent complicated infections in vulnerable groups. Unfortunately, the lack of routine hepatitis A, D, and E molecular detection in the Mexican health system means that most acute infections are missed, and timely information regarding the virus circulation is not obtained. Additionally, there is room for improvements in the hepatitis A and B vaccination coverage, which, inevitably, will impact the health of millions of at-risk people. As summarized in Table 1, many aspects of the chain of actions involved in the steps toward eliminating hepatitis viruses are windows of opportunity that need attention.

Educating health professionals and the population is imperative to controlling virus dissemination. International collaboration and access to generic medicines offer avenues for improving treatment coverage and effectiveness. Addressing the challenges and seizing opportunities for reducing the impact of viral hepatitis in Mexico entail resource allocation, expanded diagnosis and treatment coverage, and multisectoral participation in elimination initiatives. Lessons learned from the COVID-19 collaboration between the government and the private sector can inform and strengthen viral hepatitis elimination efforts.

This complex national task within the international framework also involves dedicating resources for research, establishing policies and legislation, and fostering agreements between governmental, academic, scientific, and social groups interested in eliminating hepatitis.

## Data Availability

Not applicable.
